# Polymorphic Self-Poisoning in the Isothermal Crystallization of Thermoplastic Polyurethanes

**DOI:** 10.1021/acs.macromol.5c02761

**Published:** 2026-01-08

**Authors:** Zakarya Baouch, Irene Guardincerri, Katalee Jariyavidyanont, Leire Sangroniz, Yunxiang Shi, Elmar Pöselt, Alejandro J. Müller, René Androsch, Dario Cavallo

**Affiliations:** † Department of Chemistry and Industrial Chemistry, 9302 University of Genoa, Via Dodecaneso 31, 16146 Genoa, Italy; ‡ Interdisciplinary Center for Transfer-oriented Research in Natural Sciences (IWE TFN), Martin Luther University Halle-Wittenberg, 06099 Halle/Saale, Germany; § POLYMAT, Department of Polymers and Advanced Materials: Physics, Chemistry and Technology, Faculty of Chemistry, University of the Basque Country UPV/EHU, Paseo Manuel de Lardizábal, 3, Donostia-San Sebastián 20018, Spain; ∥ BASF Polyurethanes GmbH, A30, Elastogranstraße 60, 49448 Lemförde, Germany; ⊥ IKERBASQUE, Basque Foundation for Science, Plaza Euskadi 5, Bilbao 48009, Spain

## Abstract

Thermoplastic polyurethanes (TPUs) are multiblock copolymers whose properties are strongly influenced by the crystallization of the hard segments (HS). Crystallized HSs based on 4,4′-methylenediphenyl diisocyanate/1,4-butanediol can develop two distinct polymorphs: the thermodynamically stable triclinic Form II or the kinetically favored paracrystalline Form I, each associated with different mechanical responses. While the effect of cooling rate on polymorphic crystallization has been studied, the isothermal crystallization kinetics of TPUs with varying HS content are less explored. Here, we investigate the isothermal crystallization of TPUs containing 29–80 wt % HS using differential scanning calorimetry (DSC), wide-angle X-ray diffraction (WAXD), polarized light optical microscopy (PLOM), and fast scanning calorimetry (FSC). TPUs with low HS content (29 and 33 wt %) crystallize exclusively in Form I, and the overall crystallization rate decreases monotonically with increasing temperature at low supercooling. In contrast, TPUs with ≥50 wt % HS display a nonmonotonic temperature dependence: the overall crystallization rate first increases with supercooling, then passes through a relative minimum, and rises again at larger supercooling. Structural analyses confirm that this inversion of the temperature coefficient of the crystallization rate originates from the competition between the formation of the two polymorphs. In agreement with previous literature, the rate minimum is tentatively attributed to polymorphic self-poisoning, in which Form I temporarily hinders the crystallization of Form II. These findings establish a direct link between polymorphic competition and crystallization kinetics in TPUs, providing new insights into structure formation and strategies for tailoring their properties.

## Graphical abstract

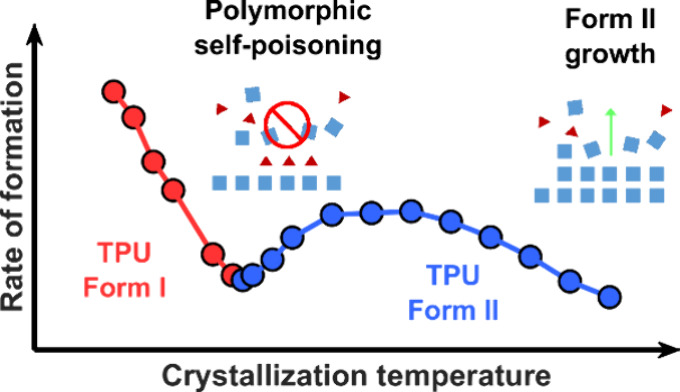

## Introduction

Thermoplastic polyurethanes (TPUs) are an important class of polymers with wide applications across various fields, mainly due to their tunable mechanical properties.^1,2^ They are composed of a multiblock arrangement of soft and hard segments (denoted as SS and HS, respectively), which typically phase-separate^3–7^ driven by crystallization, and dictate the final properties of the material, depending on their ratio.^8,9^ The SS is usually made of polyether or polyester macrodiols, with a low glass transition temperature. At the same time, the HS are rigid units resulting from the reaction of diisocyanate and short diols or diamine, yielding urethane bonds. The TPUs employed in this study have been extensively investigated in the literature.^10–29^ Their SS macrodiol is poly­(tetramethylene oxide) (PTMO) and the HSs are constituted by 4,4′-methylenediphenyl diisocyanate/1,4-butanediol (MDI/BDO).

In this system, the HSs are semicrystalline and can form distinct crystalline structures from the melt. More specifically, the triclinic Form II modification^12–15^ is the thermodynamically favored structure that forms at low supercooling or during slow cooling.^21,24,25,27^ The application of larger supercooling or higher cooling rates, instead, leads to the structuring of the HS into a paracrystalline Form I,^15,21,24,25^ which is kinetically favored and melts at lower temperatures with respect to Form II. Given that the two polymorphs exhibit different mechanical properties,^21,26,28^ understanding of the detailed formation conditions of each structure is essential.

In a previous publication,^27^ we described how to tune the polymorphic crystallization by varying the cooling rate in TPUs with varying HS content. It was shown that for HS content below 50 wt %, Form II cannot form, regardless of the applied cooling rate. On the other hand, a mixture of Form II and Form I was obtained in samples with higher HS content at cooling rates between 3 and 20 K/min, where the fraction of the more stable polymorph increases with decreasing cooling rate. Higher cooling rates initially result in the exclusive formation of Form I, until crystallization of the HS is completely suppressed. Interestingly, the polymorphic outcome, for a given TPU and cooling rate can be significantly altered by self-nucleation, with lower self-nucleation temperatures enabling the formation of larger amounts of Form II.^29^

Despite a few studies reported on the topic,^21,24,25^ the isothermal crystallization conditions leading to one or the other structure, particularly in TPUs with varying HS content, remain insufficiently investigated. This is particularly true with respect to the kinetics of polymorphic crystallization, for which, to the best of our knowledge, only a single study has been reported.^25^ In their work, Liu et al. first identified the crystallization temperature range of Form I (large supercooling) and Form II (low supercooling) development. Subsequently, they determined the overall crystallization rate via differential scanning calorimetry.^25^ Intriguingly, the trend of the crystallization kinetics versus temperature is nonmonotonic, going through a maximum with decreasing temperature, descending toward a relative minimum, and getting faster again at larger supercooling. The authors identified the predominant structure forming at each temperature: Form II in the high-temperature branch of the curve, down to the relative minimum; Form I in the low-temperature ascending branch; and a mixture of the two polymorphs at the relative minimum rate. However, the reason for this peculiar kinetic effect (a decrease in the crystallization rate with increasing temperature to a relative minimum well above the glass transition) was not discussed in detail. Incidentally, the same scheme of structure formation, giving the two pure forms at low and high supercooling and the concomitant formation of both at intermediate values, was also confirmed, without a kinetic investigation, by analysis of the micrometer-scale morphology^24^ (since Form II crystallizes in spherulites and Form I in nonbirefringent aggregates).

The scope of the present work is thus to extend the study of TPUs isothermal crystallization kinetics over a temperature range encompassing the formation of both polymorphs, accounting also for the effect of varying HS content. By combining calorimetric and structural analysis techniques, we aim to verify the peculiar temperature dependence of the overall crystallization rate and elucidate how the competition between the two polymorphs influences the solidification of the amorphous phase.

## Materials

The TPUs of the present work were produced by BASF Polyurethanes GmbH (Lemförde, Germany) using a one-shot process. The raw materials were sourced from BASF. These TPUs consist of 4,4′-methylenediphenyl diisocyanate and 1,4-butanediol, which form the hard segments. The soft segments are composed of poly­(tetramethylene oxide) macrodiol with a number-average molecular weight (*M*_n_) of about 1000 g/mol and a polydispersity index of around 2. The TPU casts were ground into chips and then injection molded to create uniform sheets for testing. Before any measurement, these sheets were annealed at 100 °C for 20 h, a common procedure to enhance the phase separation between HS and SS. Table 1 presents the composition and further relevant properties of the TPU samples analyzed in this study.

**Table 1:** Composition and Molecular Properties of the Studied TPUs

sample code	HS content (%)	MDI-BDO avg. length (number of repeat units)	*M*_w_ (g/mol)	*M*_n_ (g/mol)	polydispersity index (PI)
TPU29	29.6	2.2	99,000	45,000	2.2
TPU33	33.2	2.3	101,000	45,000	2.2
TPU50	50.0	3.7	98,000	43,000	2.3
TPU60	60.0	5.0	97,000	43,000	2.3
TPU70	70.0	7.6	93,000	41,000	2.3
TPU80	80.0	10.8	83,000	37,000	2.2

## Methods

### Differential Scanning Calorimetry (DSC)

DSC analyses were performed using a DSC250 instrument (TA Instruments, Newcastle, Delaware, USA) under a nitrogen atmosphere at a flow rate of 50 mL/min. Temperature and heat flow signals were calibrated using a high-purity indium reference. We note that thermal stability is a crucial issue when analyzing MDI/BDO-based thermoplastic polyurethanes.^30,31^ To limit degradation effects, a fresh sample of around 8 mg was used for each crystallization experiment, and exposure at elevated temperatures was restricted to a short time (1 min).^27,29^ The thermal protocol applied in DSC experiments is illustrated schematically in Figure S1a.

### Fast Scanning Chip Calorimetry (FSC)

FSC experiments were conducted with a Flash DSC 2+ (Mettler-Toledo, Greifensee, Switzerland), using conditioned and temperature-corrected UFS 1 sensors. The instrument was connected to a TC100 intracooler (Huber, Offenburg, Germany), assuring a predefined sample-support temperature of −90 °C, allowing for fast cooling and subambient temperature operation. To prevent thermo-oxidative degradation of the samples and icing of the sample compartment, the sample environment was purged with nitrogen gas at a flow rate of approximately 35 mL/min. Specimens of about 10 μm thickness were manually cut from the as-received injection-molded parts and then reduced in their lateral width to 50–100 μm, employing a scalpel and a stereomicroscope. To enhance heat transfer and minimize mechanical distortion of the sensor membrane due to thermal expansion of the polymer during measurement, we employed silicon oil and a thin layer of gold leaf as the contact medium between the sensor membrane and the specimen.

For observing the microstructure of FSC samples after specific crystallization routes, samples of a laterally larger size were prepared, omitting oil and gold leaf as a contact medium for the sake of improved imaging. We employed an Eclipse LV 100N pol microscope (Nikon GmbH, Düsseldorf, Germany), operated in reflection mode, and using polarized light. A Nikon DS-Fi3 camera was attached to the microscope for image capture.

### Polarized-Light Optical Microscopy (PLOM)

An Olympus BX-51 optical microscope (Olympus, Tokyo, Japan) equipped with a Linkam THMS 600 hot stage (Linkam Scientific, Tadworth, UK) connected to a liquid nitrogen vessel was used for in situ isothermal crystallization studies and morphological observations. The TPU80 sample was placed between two glass slides and heated to the molten state at a rate of 20 K/min up to 270 °C. It was held at this temperature for 1 min to eliminate any previous thermal history. Subsequently, the sample was cooled at 50 K/min to the selected crystallization temperature and held isothermally for a sufficient period to allow complete crystallization.

### Wide-Angle X-ray Diffraction (WAXD)

WAXD measurements were performed using a MiniFlex diffractometer (Rigaku, Tokyo, Japan) equipped with a Cu Kα X-ray source (λ = 0.154 nm). The measurements were conducted in a θ/2θ scanning mode. One-dimensional (1D) scattering curves were obtained using SmartLab Studio software. The scanning parameters were set as follows: a start angle of 5°, an end angle of 40° of 2θ, a step size of 0.05°, and a scanning speed of 2.5°/min. The X-ray generator was operated at a voltage of 40 kV and a current of 15 mA. Sample preparation was carried out in the DSC by cooling the samples from the appropriate maximum temperature to various chosen crystallization temperatures and allowing for the complete crystallization (according to the thermal protocol of Figure S1a). A sample weight of approximately 12 mg was used, and the samples were manually removed from the aluminum pans before the WAXD measurements. The description of pattern analysis to derive polymorph content estimation is reported in the Supporting Information.

## Results and Discussion

Figure 1 presents isothermal DSC crystallization curves for a selected sample (TPU80) at various temperatures. The analogous results for the other TPUs are reported in the Supporting Information, Figure S2. The data reveal a peculiar temperature dependence of the crystallization kinetics. At low temperatures (133–146 °C), crystallization begins quickly, with exothermic peaks appearing within approximately 1–5 min and with the crystallization peak time increasing with *T*_c_. However, as the temperature increases beyond 146 °C, the temperature dependence of the time to peak reverses, with the characteristic crystallization peak time decreasing with temperature, reaching a clear relative minimum at about 163 °C. Above this relative maximum in the crystallization rate, the process slows down again with increasing temperature, with the exothermic peak shifting toward longer times as the temperature approaches 183 °C.

**Figure 1:**
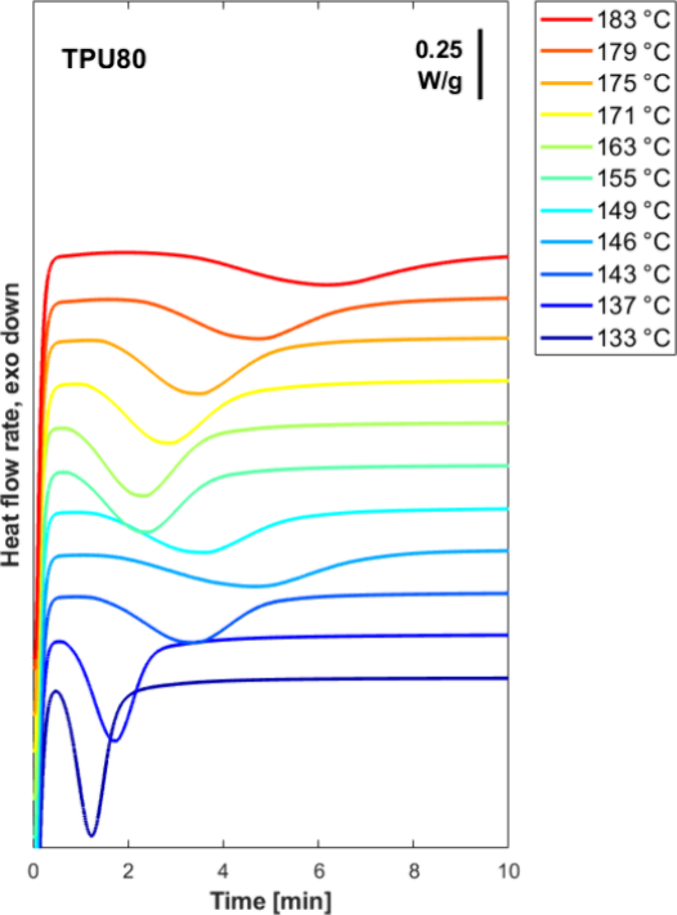
Representative DSC curves showing the evolution of heat-flow rate as a function of time at the different indicated crystallization temperatures for TPU80.

Therefore, a nonmonotonic trend in the overall crystallization kinetics versus temperature, with a relative minimum at 146 °C and a maximum at 163 °C, is highlighted in Figure 1. Similar behavior, however, with the minimum in crystallization rate occurring at different temperatures, is reported with Figure S2 for the samples containing 50–70 wt % HS (Figure S2c–e). In contrast, a “standard” monotonic decrease in crystallization kinetics with increasing temperature is observed for TPU29 and TPU33 (Figure S2a,b). The presence of a relative minimum in the crystallization kinetics as a function of temperature can be explained by different reasons, including: (i) a transition between heterogeneous and homogeneous nucleation mechanisms;^32,33^ (ii) the temperature-dependent formation of different polymorphs with different growth rates, which triggers a self-poisoning effect of the metastable structure on the more stable one.^34–36^ These possibilities will be discussed in detail below.

From isothermally recorded DSC curves, the time to the exothermic peak in the heat-flow rate can be extracted to quantitatively assess the overall crystallization kinetics (including both primary nucleation and growth) of the samples at different temperatures. The overall crystallization rate of a polymer is often characterized by the reciprocal of the half-crystallization time or of the time to peak from isothermal crystallization experiments.^37^ Therefore, Figure 2 collects these values for the different TPUs at the analyzed crystallization temperatures. The reciprocal of the time to peak is chosen, rather than that of the half-crystallization time, due to its easy determination for all the experiments, even in those at low crystallization temperatures (for which the half-crystallization time is difficult to determine because of the nonsteady state instrumental heat-flow rate).^38^

**Figure 2:**
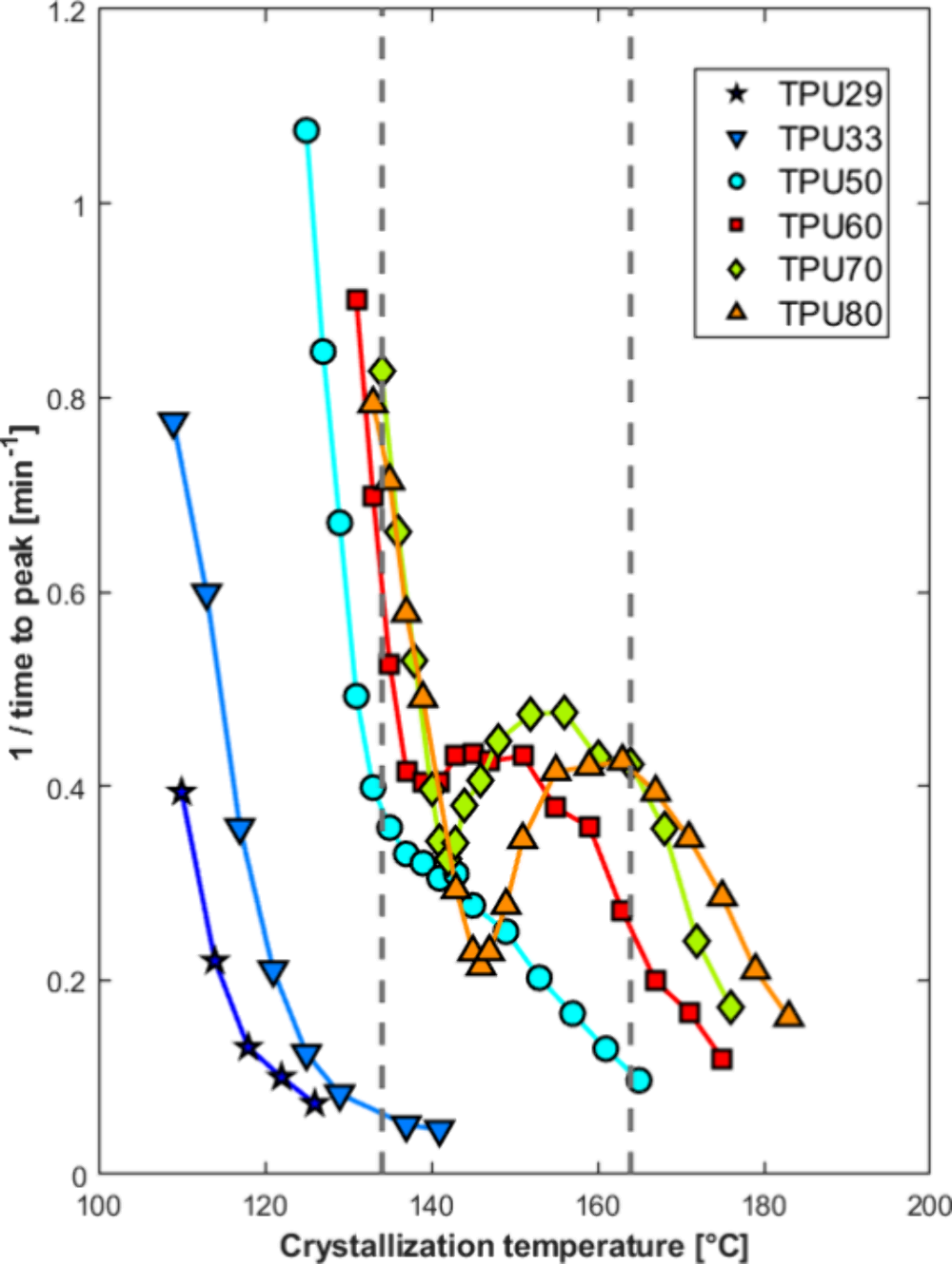
Reciprocal of crystallization peak time as a function of crystallization temperature for different TPU samples. The dashed vertical lines represent the crystallization temperatures considered for further analysis (Figure S3a).

Starting from the TPUs with low HS content, i.e., TPU29 and TPU33, a monotonic decrease in the overall crystallization rate with increasing temperature is observed over the analyzed temperature range, in agreement with the expectation of a decrease in the crystallization rate as the equilibrium melting temperature is approached. TPU33, which has a higher content of crystallizable hard segments, appears to exhibit a higher maximum crystallization rate than TPU29, though the analyzed temperature range is too narrow for a final conclusion. It is worth noting that these two samples are the only ones unable to develop Form II crystals upon nonisothermal crystallization conditions, as evidenced in a previous work.^27^ In fact, they crystallize exclusively into Form I crystals even at low cooling rates, while a meaningful content of Form II could be produced only by applying self-nucleation.^29^

Instead, when the HS content reaches and exceeds 50 wt %, the temperature dependence of the overall crystallization rate becomes nonmonotonic. From high to low temperature, the crystallization rate first increases, reaches a more or less pronounced maximum (for TPU60, TPU70, and TPU80), decreases to a relative minimum of varying depth, and eventually increases again. In the case of TPU50, no pronounced relative minimum of the crystallization rate is detected; instead, a clear inflection point appears at an intermediate temperature, distinguishing a low- and a high-supercooling branch of the curve.

An analogous temperature-dependence of the crystallization kinetics, displaying an evident relative minimum with temperature, has been reported for a TPU of unspecified HS content by Liu et al.^25^ In that case, investigation of the structure confirmed the formation of the Form I polymorph in the low-temperature branch of the curve, and of Form II crystals at temperatures encompassing the relative maximum. The reason behind the occurrence of the relative minimum in kinetics was not discussed in detail in their work. Assuming that the same temperature-dependent polymorphic outcome applies to the data of Figure 2, the nonmonotonic overall crystallization rate curves resemble known cases of polymorphic self-poisoning reported by Alamo et al., for different dimorphic polymer systems.^35,36^

From Figure 2, quantitative information to compare the dependence of specific features on the hard segment content of the TPUs are reported in Figure S3 of the Supporting Information.

To confirm the assignment of the different branches of the overall crystallization rate temperature plot to the development of different polymorphs, WAXD analysis of isothermally crystallized samples has been performed. As a representative example, Figure 3 shows the WAXD patterns of TPU80 recorded at room temperature after isothermal crystallization at different *T*_c_.

**Figure 3:**
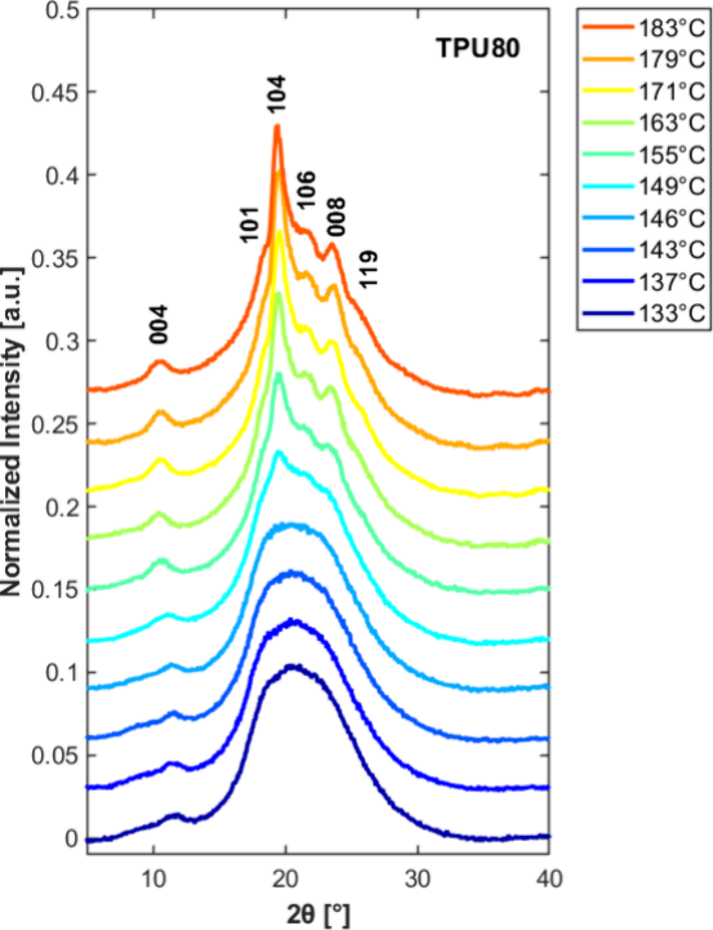
WAXD patterns of TPU80 recorded at room temperature after isothermal crystallization at the indicated temperatures. The crystalline planes indexes for Form II structure are indicated.^21,23^

The data reveal a clear evolution in crystalline structure depending on the crystallization temperature. At lower *T*_c_ values (133–146 °C), the diffractograms are dominated by a broad halo with a weak peak centered around 2θ ≈ 20.5° and a faint peak above 10°, features typically associated with Form I crystals.^25^ In fact, this paracrystalline form exhibits poor chain packing and low long-range order, resulting in broad and low-intensity reflections.^13^

As the crystallization temperature increases, the patterns become dominated by more defined diffraction peaks. In particular, on crystallization above 149 °C, the intensity of the 2θ ≈ 20° peak increases markedly, and additional peaks, most notably one around 2θ ≈ 10°, become progressively more prominent with increasing crystallization temperature. On the basis of the literature^21,23,25^ and of previous work from our group,^27,29^ these features in the diffractograms can be assigned to the development of Form II crystals, the more ordered and thermodynamically stable polymorph. According to the visual inspection of the diffraction patterns, it can be deduced that the Form II crystal content increases with *T*_c_.

An analogous behavior was observed for the samples with varying HS content in the range 50–70 wt % (see Figure S4), with a noticeable decrease in the intensity of Form II diffraction peaks for the polymers containing fewer/shorter hard segments. Notably, no change in structure with crystallization temperature is observed for the sample containing lower HS content, namely TPU29 and TPU33. We underline that these samples have also been shown to be unable to develop Form II crystals upon cooling at slow rates (<1 K/min), due to the occurrence of thermal degradation.^27^

The observed switch from Form II at higher *T*_c_ to Form I at larger supercooling reflects a common behavior in polymorphic polymers, where thermodynamics favors the stable form at high temperatures, while increasing supercooling progressively shifts the kinetics toward growth of the metastable form.

Once the general trend of the WAXD patterns of isothermally crystallized TPUs has been discussed, it is useful to quantitatively determine an index representative of the polymorphic content of the sample. As in previous work, we analyzed the WAXD intensity of the 104 peak of Form II crystals (after intensity normalization for each pattern), which is practically absent when only Form I crystallizes, and increases with the content of Form II. Figure 4 compares the crystallization temperature dependence of this Form II peak WAXD intensity (red points) with the crystallization kinetics determined via DSC (reciprocal of the time to peak, blue points) for different selected TPUs with varying HS content.

**Figure 4:**
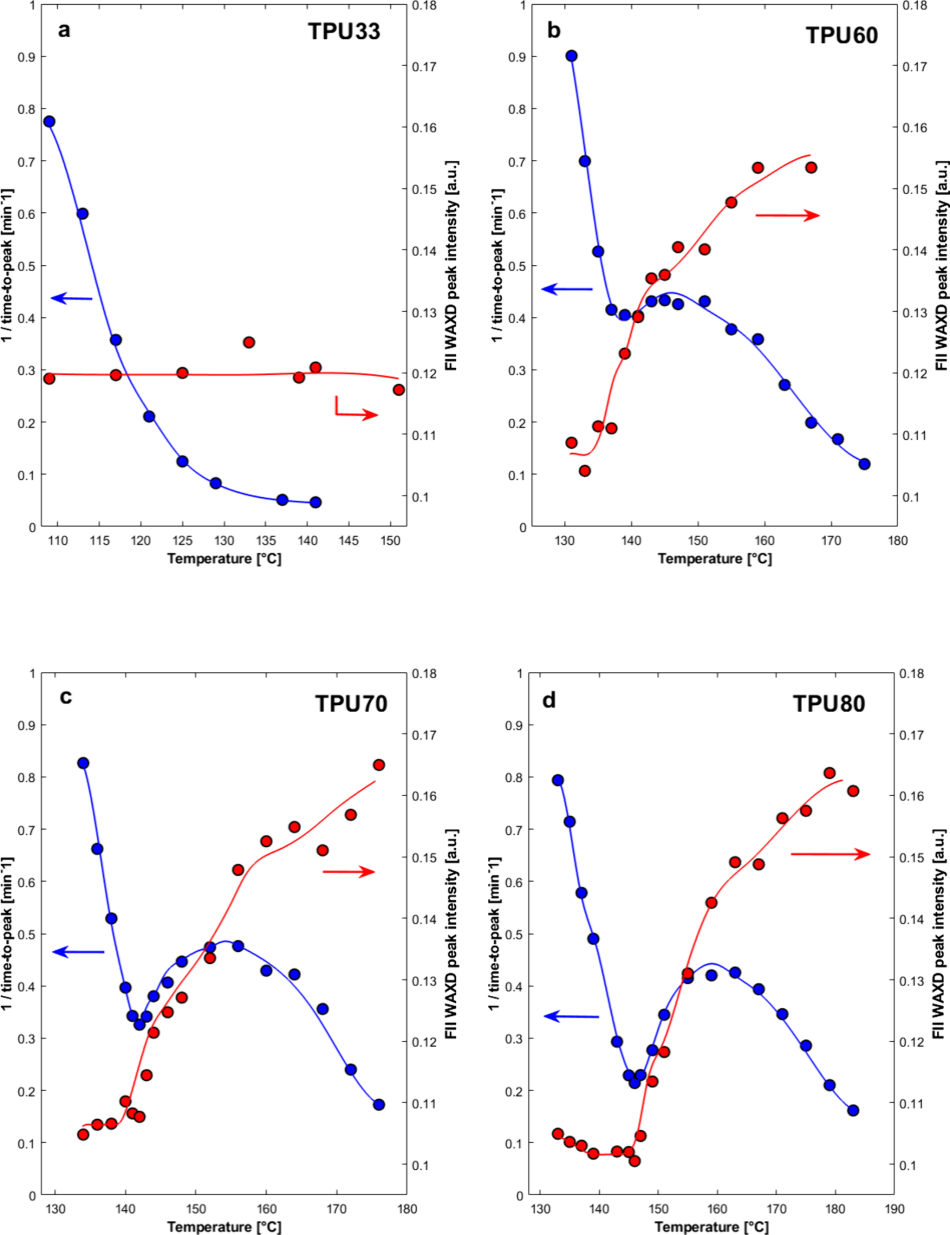
Comparison of reciprocal of crystallization peak time from DSC and Form II (FII) WAXD intensities at approximately 20° (2θ), as a function of crystallization temperature for TPU33 (a), TPU60 (b), TPU70 (c), and TPU80 (d). The continuous lines are a guide to the eyes.

In TPU33, a smooth monotonic decrease in the overall crystallization rate with increasing temperature is observed. Accordingly, the intensity of the Form II WAXD main peak does not change with *T*_c_, as the sample crystallizes into Form I throughout the entire temperature range (see Figure S4b). This is consistent with previous investigation in nonisothermal conditions,^27^ which highlighted that Form I crystals could not develop for HS content below 50 wt %. Obviously, an analogous behavior is shown by TPU29 (Figure S5a).

For HS content above 33 wt %, i.e., Figures 4b–d and S5b, the crystallization kinetics exhibits a more or less pronounced relative minimum with increasing temperature. Interestingly, this temperature coincides with the onset of a sharp increase in Form II crystal content in the sample, suggesting a strong correlation between polymorph selection and crystallization kinetics. In the low supercooling range, the kinetics are the slowest, while the Form II crystal content is the highest. With decreasing *T*_c_, the overall crystallization rate goes through a maximum, and the Form II WAXD intensity progressively declines until reaching a minimum constant value, in the presence of the Form I polymorph only (see Figures 3 and S4 for the related WAXD patterns). In this plateau of Form I content, the crystallization kinetics display a sudden rise with decreasing temperature.

The comparison of structural and calorimetric data confirms that two different structures are formed above and below the relative minimum, as previously hypothesized. This finding suggests a polymorphic self-poisoning mechanism behind the occurrence of the relative minimum in the overall crystallization kinetics for TPU with HS content above 50 wt %.

Self-poisoning is a peculiar kinetic effect arising from the competitive crystallization of two “polymorphs” in the broader sense. It was first discussed by Ungar and Keller for the crystallization kinetics of long-chain *n*-alkanes.^39–43^ In this case, an inversion of the temperature dependence of the crystallization rate with the occurrence of a relative minimum with increasing supercooling was observed. The finding was interpreted as the result of a competition between extended-chain and folded-chain crystals formation. In particular, as temperature decreases, the attachment of folded-chain stems at the growth front of extended-chain crystals retards the growth of the latter, until, with sufficient supercooling, the folded-chain crystals can eventually grow faster. Later on, similar effects were discussed in detail also for a “truly” polymorphic system, i.e., isotactic polystyrene in decalin solution,^44^ where crystals with different helical and trans-planar chain conformations are obtained in different supercooling regions. Since then, polymorphic self-poisoning has also been recognized in small organic molecules^45^ and other polymers.^35,36^ Recently, modeling of the self-poisoning effect on the crystallization kinetics in poly­(ethylene brassylate)^46^ and molecular dynamic simulations of polymorphic competition with supercooling in bromo-substituted polyethylene^47^ have also been reported.

Accordingly, for the analyzed TPUs, it can be hypothesized that, as the rate of Form I crystallization becomes faster and faster with decreasing crystallization temperature (below its equilibrium melting temperature), the Form II development becomes progressively more and more hindered by the attachment of Form I nuclei on the growth front of Form II crystals. Before the growth of Form II can proceed, such Form I nuclei must first detach, leading to a slowing down of the crystallization rate of the stable polymorph Form II, in agreement with the self-poisoning mechanism.^48,49,35,36^ In the specific case of thermoplastic polyurethanes, it should be noted that cross-nucleation between the two polymorphs has been reported. Based on the analysis of the polarized light optical micrographs obtained at different crystallization temperatures, both Form II nucleating on the periphery of Form I crystals and the opposite phenomenon are observed.^24^ This literature result provides morphological evidence about the possibility that Form I hinders Form II crystallization at intermediate supercooling, where the two structures kinetically compete, thus giving support for the “molecular” self-poisoning interpretation.

It is important to note that the reported slowing down of the kinetics of Form II formation occurs far away from the glass transition temperature of the polymer, and can not thus be attributed to diffusion limitation, also because by further lowering the temperature, the metastable polymorph again crystallizes faster. The proposed hypothesis of polymorphic self-poisoning to justify the observed nonmonotonic crystallization kinetics of TPUs aligns with previous investigations on dimorphic precision polyethylenes with bromine and a polyacetal from Alamo and co-workers,^35,36^ where this effect was reported and thoroughly discussed. Moreover, as reviewed by Cavallo et al.,^34^ even if seldom discussed in these terms, the occurrence of relative minima in the crystallization or growth rate versus temperature is frequently observed during concomitant/competitive crystallization of two polymorphs. We underline that the TPU polymorphs possess different conformations of the 1,4-butanediol segments in the crystal structure,^12,14^ namely more contracted for the triclinic polymorph Form II and more extended for the metastable one (Form I). As such, the proposed self-poisoning effect in the TPU could have an analogous origin, i.e., a mismatch in molecular conformations of the polymorphs at the growth front, to that observed in long-chain *n*-alkanes^39–43^ (extended versus folded chain), polyethylene with precisely spaced bromine atoms^35^ (herringbone versus zigzag) or polyacetal with 12 methylene sequences between the acetals group^36^ (increase in lamellar thickness by one repeating unit).

Given the fact that self-poisoning in polymer crystallization is commonly manifested trough the occurrence of a sharp minimum in crystal growth rates as a function of temperature,^35,36,41,50,51^ and although nucleation rates^41^ (and hence overall transformation rates^35,36,39,40,42,43,50,51^ ) can also display such minimum, in the following we discuss PLOM measurements carried out on one of the TPU samples exhibiting a deep relative minimum in the temperature dependence of the overall crystallization kinetics.

Figure 5 presents PLOM micrographs of TPU80 crystallized isothermally at three representative temperatures located in different regions of the overall crystallization rate curve: 146 °C (at the rate relative minimum), 155 °C (intermediate temperature, around the relative maximum), and 183 °C (high-temperature branch).

**Figure 5:**
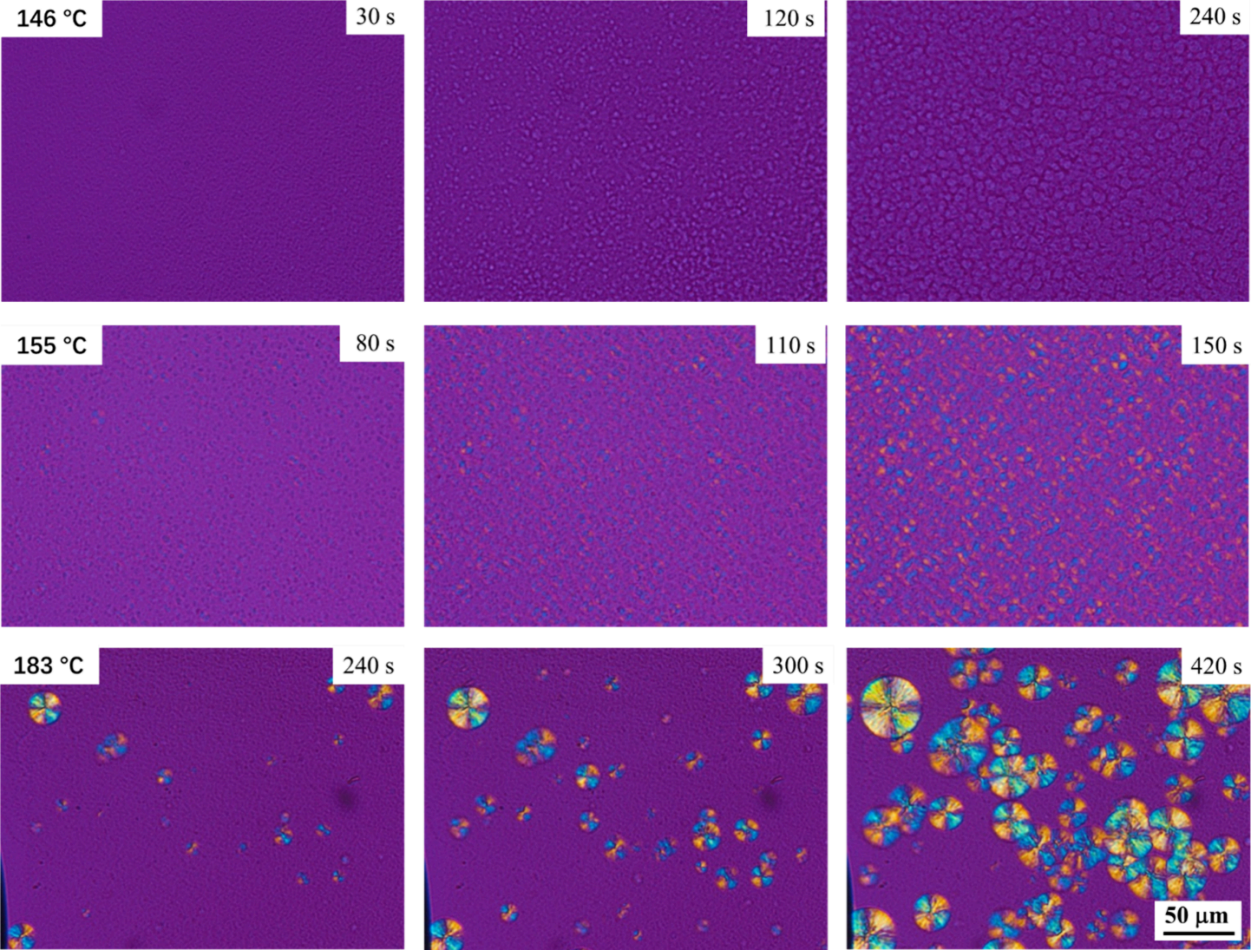
Polarized light optical micrographs acquired at different times during crystallization of TPU80 at the indicated isothermal temperatures.

At 146 °C, the sample crystallizes in the form of nonbirefringent crystals, which grow until impingement. This morphology, lacking birefringence, is characteristic of the Form I structure,^21,24^ possibly due to “pseudo-flat-on” arrangement of the crystals with respect to the microscope glass plane^24^ or to the short coherence length of the crystals, below the wavelength of light. We note that the relatively high number of nonbirefringent crystals prevents an easy determination of their growth rate. Notably, at the temperature of the minimum overall crystallization rate, no birefringent spherulites, indicating the presence of Form II, are detected.

At 155 °C, around the relative maximum of the overall crystallization rate, faint birefringent structures, attributed to Form II, become visible over time, appearing as very small negative spherulites. On the other hand, especially at short times, nonbirefringent regions corresponding to Form I crystals are also visible. The concomitant formation of these two morphologies has already been reported for segmented polyurethanes at moderate supercooling.^24^ Due to this particularly dense dual morphology, any quantification of the growth rates of the two polymorphs is not possible.

Well-developed, birefringent spherulites displaying clear Maltese-cross patterns dominate the morphology at 183 °C. At low supercooling, the thermodynamic conditions favor the exclusive formation of Form II crystals, albeit at a decreasing rate with increasing *T*_c_. The size and number of the spherulites allow a quantification of the growth rate of Form II, although only in this particular temperature range. The measured growth rates ranges from about 3.6–5.4 μm/min for temperatures going from 183 to 171 °C, respectively.

The morphological observations at three meaningful crystallization temperatures support the conclusions on the temperature-dependent polymorphism inferred from WAXD (Figures 3 and S4) and kinetics (Figures 2 and 4) analyses. Moreover, the hypothesized polymorphic self-poisoning is also compatible with the morphology, as in the temperature range where the overall crystallization kinetics are slowed down by decreasing *T*_c_, i.e., from the relative maximum downward, the concomitant formation of the two polymorphs and their competition for structuring the amorphous phase are observed (see Figure 5, *T*_c_ = 155 °C).

Despite this, since the growth rate is not directly measurable in an extended temperature range in these TPUs, the interpretation of the relative minimum in the crystallization kinetics as a self-poisoning effect must rely on the overall crystallization rate data by DSC only. While extensive literature on the effect of self-poisoning on growth rate both in bulk and solution exist,^35,36,41,50–56^ there are also some works discussing the issue of self-poisoning in different systems based on the overall crystallization rate alone, such as in solution crystallization of long-chain alkanes^39,40,42,43^ and in melt crystallization of an aromatic polyketone.^57^

To expand the supercooling interval achieved via DSC and PLOM, FSC experiments were carried out on selected samples. In particular, we focus on two samples: one displaying a well-evident relative minimum in the overall crystallization rate (TPU80) and another that only exhibits a monotonic decrease in the crystallization kinetics with increasing temperature (TPU33) (see DSC data in Figure 2). Figure 6 shows, as an example, representative fast-scanning calorimetry heating curves collected after isothermal crystallization of TPU80 after selected holding times at two crystallization temperatures, 120 °C (a) and 155 °C (b). As outlined by the above-discussed results (particularly WAXD, Figures 3 and 4), these two crystallization temperatures correspond to predominant development of Form I (120 °C) and Form II (155 °C), respectively. In fact, distinct melting behavior can be inferred after crystallization at the two temperatures.

**Figure 6:**
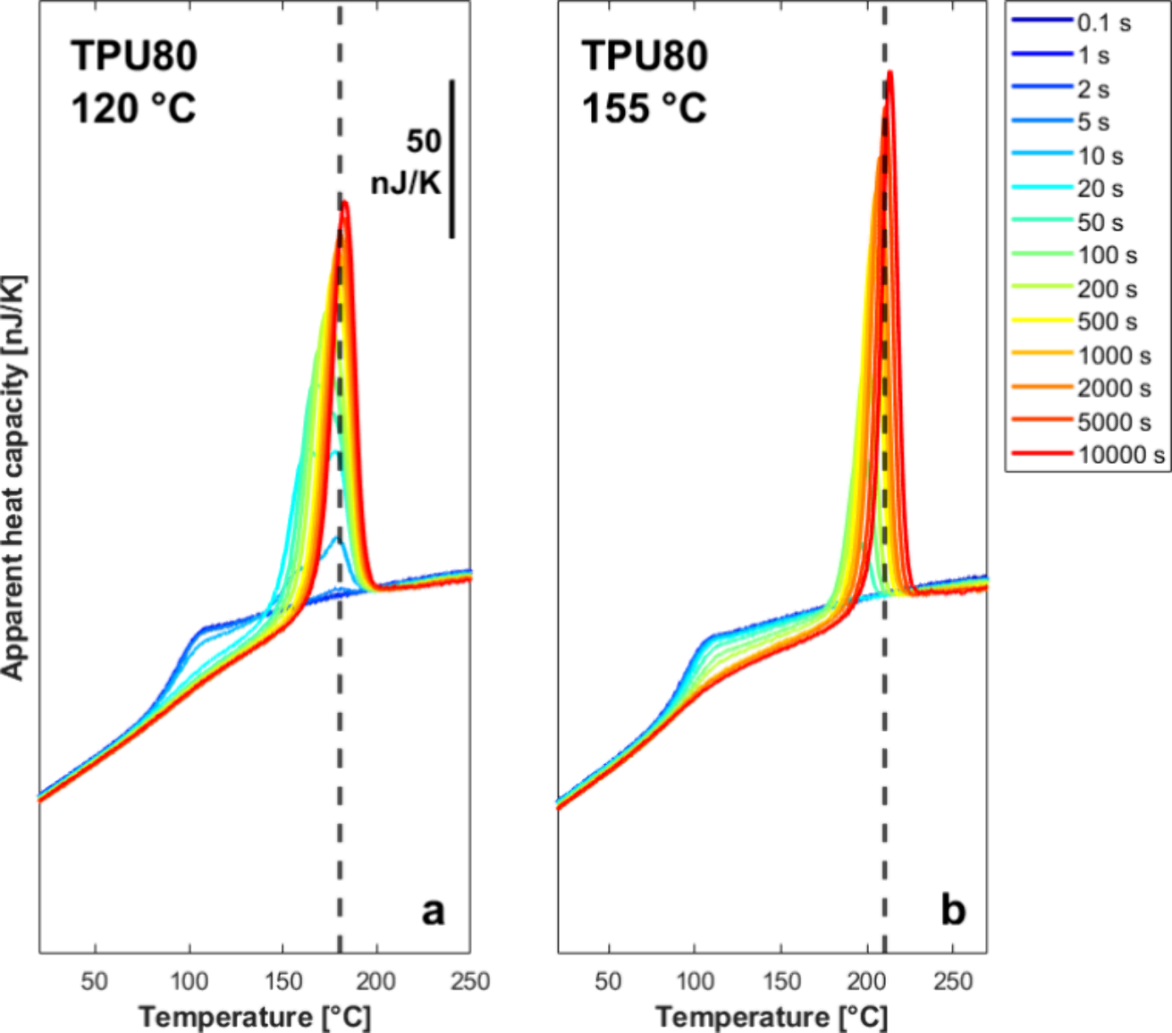
Apparent heat capacity as a function of temperature during heating TPU80 after isothermal crystallization at the indicated times. Samples were crystallized at 120 °C (a) and 155 °C (b). Dashed vertical lines are added at temperatures of 180 °C (a) and 210 °C (b) to help the reader estimating the peak melting temperature.

At the high FSC heating rate adopted (1000 K s^–1^), the melting endotherm appears as a single, well-defined peak. This is particularly true for Form II crystals formed at 155 °C (Figure 6b), while Form I crystals grown at 120 °C reveal double melting at lower crystallization times (Figure 6a). This behavior is in contrast with the usual complex melting behavior of TPU, displaying multiple peaks typical of melting–recrystallization phenomena, commonly observed in conventional DSC.^25,58–61^ This is expected as fast heating in FSC experiments effectively suppresses crystal reorganization during scanning,^17^ allowing to detect the melting of the original crystal population.

By considering the melting endotherms at increasing isothermal holding time, for the two crystallization temperatures selected, three clear trends are evident: (i) the total melting enthalpy increases steadily with time, indicating the increase of the crystalline fraction; (ii) the melting peak temperature shifts to higher temperature, consistent with a progressive perfectioning and increased thermal stability of the formed crystals with longer isothermal crystallization; (iii) the magnitude of the step change in apparent heat capacity at the glass transition progressively decreases with increasing holding time, reflecting the reduction of the mobile amorphous fraction as crystallinity develops. We also note that, besides the evident thermal stability difference between the two polymorphs (i.e., increase of melting temperature by about 30 K for Form II crystals with respect to Form I crystals), the melting peaks formed after crystallization at 155 °C are generally sharper than those formed at 120 °C, suggesting a narrower crystallite thickness distribution for Form II crystals.

While a detailed mechanistic study of crystal perfectioning during crystallization is beyond the scope of the present work, the increase in melting enthalpy with isothermal crystallization time is used to derive information on the overall crystallization kinetics. More specifically, the melting enthalpy versus crystallization time curves (not shown) display a typical sigmoidal increase from which the half-time of crystallization is estimated. Figure 7 shows the reciprocal of this time, proportional to the crystallization rate, for TPU80 (a) and TPU33 (b), respectively. Measurements and repetitions, as well as analyses performed on several samples, are reported on the same plot to assess reproducibility. Particular care was taken to ensure that sample degradation in multiple measurements could be excluded.

**Figure 7:**
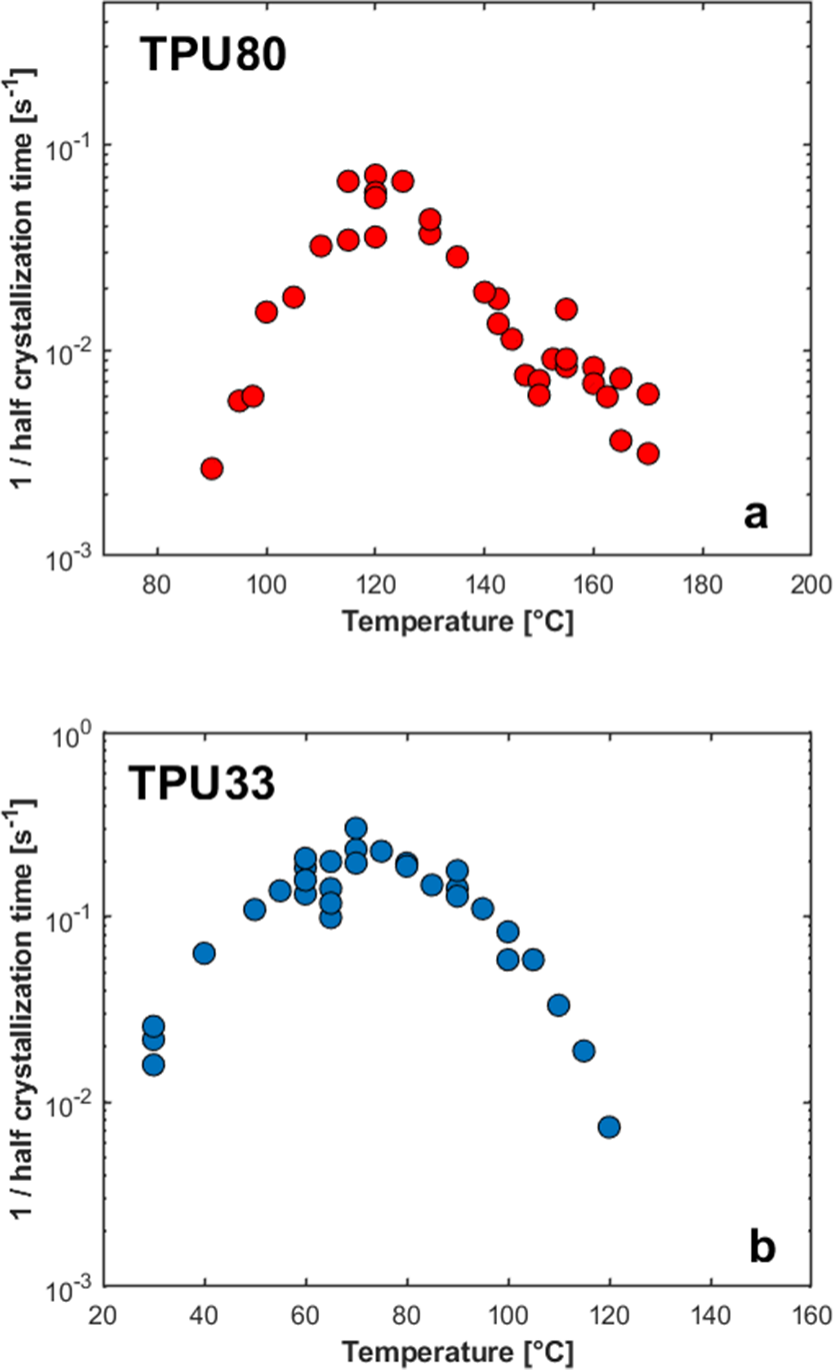
Reciprocal of the crystallization half-time measured by FSC as a function of crystallization temperature for TPU80 (a) and TPU33 (b). Several measurements are reported to show the reproducibility and absence of sample degradation.

The data reveal that TPU80 (Figure 7a) exhibits a bimodal dependence of the crystallization rate on temperature, with an absolute maximum around 120 °C and a second relative maximum at approximately 155 °C. The crossover from one bell-shaped curve to the other occurs approximately at 150 °C. In agreement with previous WAXD analysis on DSC-crystallized samples of TPU80 (Figures 3 and 4), the two curves can be associated with the predominant formation of Form II crystals (above 150–155 °C) and of Form I crystals (below 150 °C). On the other hand, for TPU33, a single bell-shaped curve of the overall crystallization rate versus temperature is observed (Figure 7b). This suggests the formation of a single polymorph, i.e., Form I, as indicated by the WAXD measurements (Figures S4b and 4a). Finally, the crystallization of the two samples occurs in a widely different temperature range. However, the absolute values of the reciprocal half-crystallization time can be compared at 120 °C, revealing that Form I crystal development in TPU33 is approximately 7 times slower than in TPU80, confirming the discussion in Figure S3a.

Thanks to the cooling rate capabilities of the FSC, the supercooling range explored via conventional DSC could be largely extended, down to temperatures close to the polymer glass transition. This allowed the recording of the expected slowing of the overall crystallization rate at low temperatures, due to limitations on chain mobility at the TPU’s glass transition temperature, i.e., around 30 and 80 °C for TPU33 and TPU80, respectively. Moreover, this confirms that the first reduction of the crystallization kinetics of TPU80 at high temperatures, corresponding to the relative minimum around 150 °C is not due to restriction to chain diffusion, as this occurs at much higher supercooling. While the possibility that the double bell-shaped kinetics is due to a change from heterogeneous to homogeneous nucleation, as frequently observed for different polymers, can not be completely ruled out, the collected WAXD and PLOM (Figures 3–5) data indicate a change in the crystallizing polymorph without a distinct increase of the nuclei number (see the PLOM image of Figure 5), compatible with the occurrence of polymorphic self-poisoning.

The FSC data reported in Figure 7 are directly compared with DSC results (Figure 2). The two sets of data are shown on the same plot in Figure S6, illustrating only minor differences, likely related to the different specifics of the employed instrumentation (effects due to sample size, substrate, temperature gradients, etc.).^62,63^

Figure 8 shows the distinct crystallization morphologies of TPU80 and TPU33, as captured by PLOM during isothermal crystallization on the Flash DSC sensor. For TPU80, two representative crystallization temperatures were chosen to probe the formation of the two polymorphs, Forms I and II. At 120 °C, corresponding to the temperature of the maximum crystallization rate associated with Form I, the morphology is featureless under polarized light, without detectable birefringence. This absence of optical contrast is consistent with the formation of Form I, as highlighted in previous work.^21,27^ In contrast, at 155 °C, corresponding to the relative maximum at higher temperatures in the crystallization rate curve, well-developed spherulites with strong birefringence and Maltese cross patterns are observed. These structures are characteristic of Form II.^21,27^ TPU80 clearly demonstrates a switch in the morphology depending on the selected isothermal crystallization temperature, directly reflecting the polymorphic outcome (Form I vs Form II).

**Figure 8:**
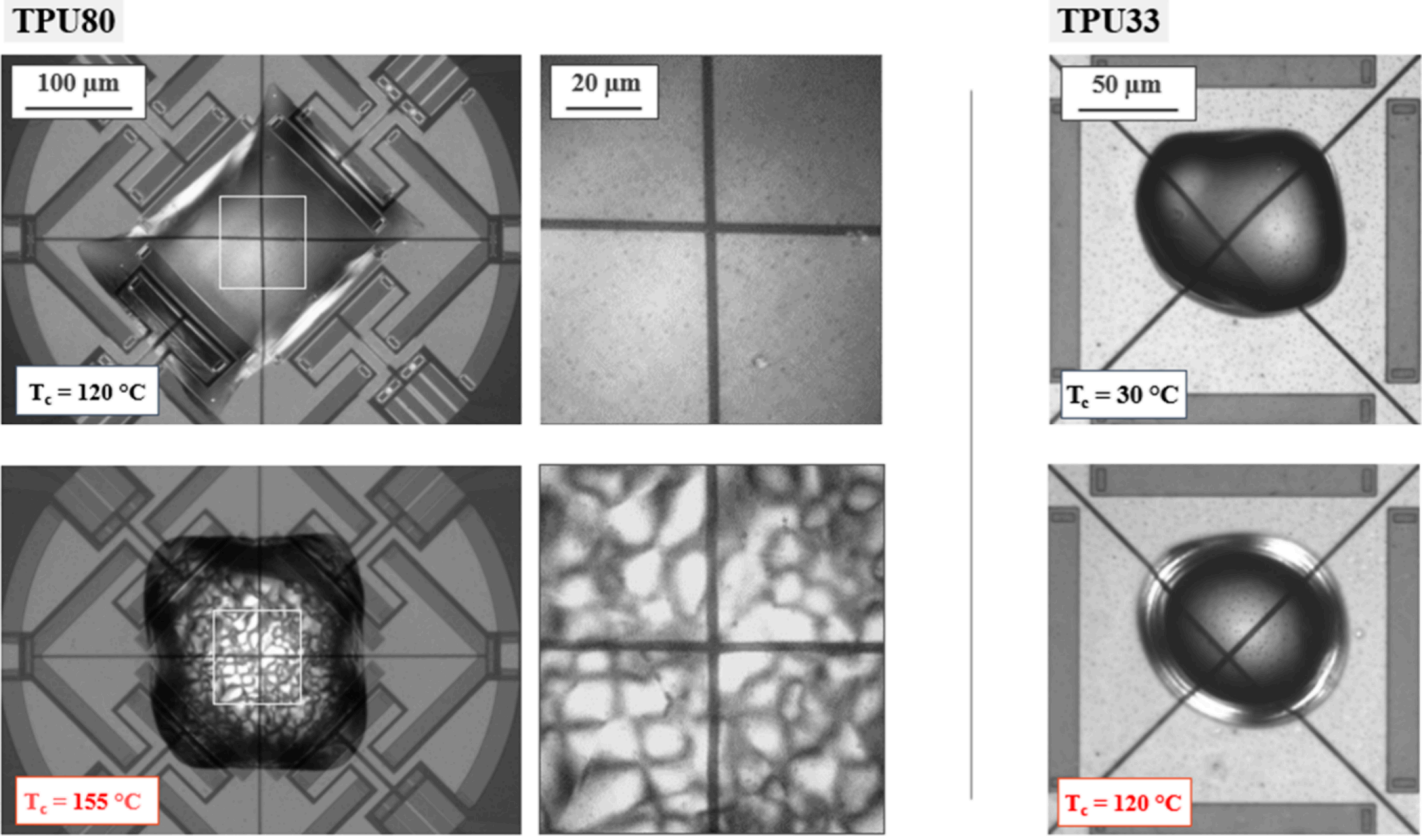
PLOM images of TPU80 (left) and TPU33 (right) isothermally crystallized on a Flash DSC sensor at the indicated crystallization temperatures. The white-boxed areas in the left images of TPU80 are shown enlarged to the right. Scale bars also hold for the corresponding lower-row images.

For TPU33, in contrast, the morphology remains consistently nonbirefringent across the entire supercooling range explored (examples are shown at 30 and 120 °C). This corroborates previous results based on crystallization kinetics and WAXD, confirming that TPU33 crystallizes exclusively in Form I, without developing the spherulitic Form II observed for TPU80. The PLOM analysis of samples crystallized on FSC sensors enables the extension of observations made via hot-stage PLOM (Figure 5) to a broader range of supercooling.

## Conclusions

Differential scanning calorimetry revealed that TPUs with relatively low HS content (29 and 33 wt %) display a monotonic temperature dependence of the overall crystallization rate, which slows down with increasing crystallization temperature, consistent with nucleation-controlled behavior. In contrast, for TPUs containing ≥50 wt % HS, the crystallization rate versus temperature exhibits a surprising nonmonotonic trend: with increasing supercooling, it first reaches a maximum, then decreases to a relative minimum, and eventually increases again at large supercooling.

Ex-situ WAXD measurements on isothermally crystallized samples confirmed that this peculiar inversion of the temperature dependence of the overall crystallization rate is associated with a change of the structure of the growing crystals: at low temperatures, the metastable Form I crystallizes; at high temperatures, the more stable Form II predominates, while at intermediate temperatures a mixture of the two structures is observed. The occurrence of a relative minimum in crystallization rate as a function of temperature, particularly clear for HS content between 60 and 80 wt % suggests a case of polymorphic self-poisoning, where the attachment of secondary Form I nuclei on the growth front of Form II crystals temporarily blocks the stable polymorph crystallization, until their detachment, thus slowing down the kinetics.

FSC results showed that the crystallization kinetics of TPU33 displayed a monomodal bell-shaped curve versus temperature, while those of TPU80 showed a bimodal curve. PLOM analysis of samples crystallized under controlled conditions on the FSC sensors enables linking the two branches of the curve for TPU80 to the different polymorphic morphologies.

Taken together, these results establish a link between the polymorphic outcome of crystallization in TPU and structuring kinetics. Specifically, they demonstrate that when the two polymorphs compete for the solidification of the amorphous phase, peculiar kinetic effects, interpreted as polymorphic self-poisoning, can arise. This detailed study, on TPUs with a broad range of HS content, provides novel insights into their crystallization behavior, which are valuable for understanding polymorphic structure formation and for tuning the properties in this class of materials.

## Supplementary Material



## Notes

Method section with DSC/FSC thermal histories, description of WAXD pattern analysis, DSC isothermal measurement for different TPU samples, analysis of polymorphs’ crystallization rate and temperature at minimum rate vs hard-segments content, WAXD patterns of different isothermally crystallized TPUs, comparison of overall crystallization and Form II content vs temperature for TPU29 and TPU50, and comparison of FSC and DSC kinetics data for TPU33 and TPU80 (PDF)
