# Supplementary material for: Polymorphic Self-Poisoning in the Isothermal Crystallization of Thermoplastic Polyurethanes
Source: Macromolecules. 2026 Jan 8;59(2):950–61. doi: 10.1021/acs.macromol.5c02761 (PMC12854736; doi:10.1021/acs.macromol.5c02761)
Supplement: Supplementary file 1 [file ma5c02761_si_001.pdf]

SUPPORTING INFORMATION

Polymorphic self-poisoning in isothermal crystallization of thermoplastic polyurethanes

Zakarya Baouch[1], Irene Guardincerri[1], Katalee Jariyavidyanont[2], Leire Sangroniz[3], Yunxiang Shi[3], Elmar Pöselt[4], Alejandro J. Müller[3,5]*, René Androsch[2]*, Dario Cavallo[1]*

[1] *Department of Chemistry and Industrial Chemistry, University of Genoa, Via Dodecaneso 31, 16146, Genoa, Italy.*

[2] *Interdisciplinary Center for Transfer-oriented Research in Natural Sciences (IWE TFN) Martin Luther University Halle-Wittenberg, 06099 Halle/Saale, Germany.*

[3] *POLYMAT, Department of Polymers and Advanced Materials: Physics, Chemistry and Technology, Faculty of Chemistry, University of the Basque Country UPV/EHU, Paseo Manuel de Lardizábal, 3, Donostia-San Sebastián 20018, Spain.*

[4] *BASF Polyurethanes GmbH, A30, Elastogranstraße 60, 49448 Lemförde, Germany.*

[5] *IKERBASQUE, Basque Foundation for Science, Plaza Euskadi 5, Bilbao 48009, Spain.*

METHODS

Differential and fast scanning calorimetry

The thermal protocol applied in DSC experiments is illustrated schematically in Figure S1a. It consists of five sequential steps specifically designed to investigate the isothermal crystallization behavior of these TPU samples:

(a) The sample was first heated to a temperature approximately 30 °C above its melting peak temperature and held for 1 minute to eliminate any prior thermal history. The selected temperatures were 250 °C for TPU29, TPU33, TPU50 and TPU60, 260 °C for TPU70, and 270 °C for TPU80.

(b) The sample was then cooled at a rate of 40 K/min to a predetermined crystallization temperature (Tc) specific to each TPU grade.

(c) Isothermal crystallization was carried out at Tc for a suitable time, which allows the completion of crystallization.

(d) After the isothermal step, the sample was cooled from Tc to 30 °C at a rate of 20 K/min. The sample was then collected for ex-situ Wide-Angle X-ray Diffraction (WAXD) analysis.

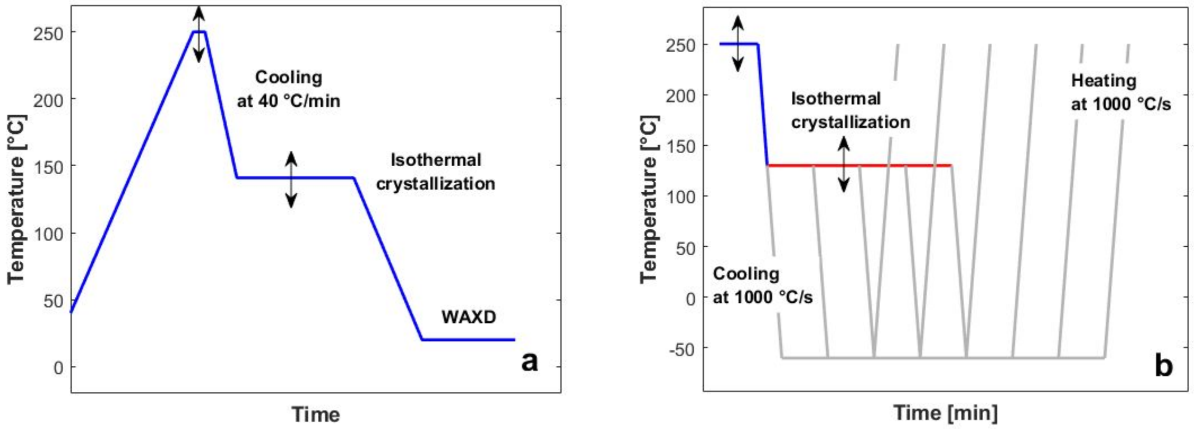

Figure S1. Schematic illustration of the thermal protocols to study the kinetics of isothermal crystallization of TPUs by DSC (a) and FSC (b).

The use of FSC enabled studying the overall isothermal crystallization kinetics of selected samples in a wider range of supercoolings, as compared to standard DSC. In order to do so, the thermal protocol described schematically in Figure S1b was adopted. The sample was heated to a temperature in the range from 250 to 270 °C, depending on HS content (see the above description of the DSC procedure) and equilibrated for a period of 0.1 s. Afterwards, the sample was cooled at 1000 K/s to the selected isothermal crystallization temperature and allowed to crystallize for a given time, before being further quenched to -60 °C and re-heated at 1000 K/s after a short equilibration time of 0.1 s The cycle is then repeated, interrupting the crystallization at various, progressively longer times. The analysis of the final heating steps allows to derive the melting enthalpy, corresponding to the amount of crystals developed during the isothermal crystallization stage.

Wide Angle X-ray Diffraction

To provide a rough estimate of the Form II polymorph content, the maximum intensity of a selected diffraction peak of this structure has been extracted. The intensity calculation includes, at first, the subtraction of a linear baseline between 5° and 40° 2θ, followed by normalizing the total area of the pattern in the same angular range to unity, neglecting the different scattering power of amorphous and crystalline regions when comparing samples of different crystallinity. From these normalized diffractograms, the maximum intensity in the angular range from 18.8 to 20.8° 2θ is obtained, that is, in the region of the emergence of the Form II peak of interest.

RESULTS

Isothermal crystallization kinetics

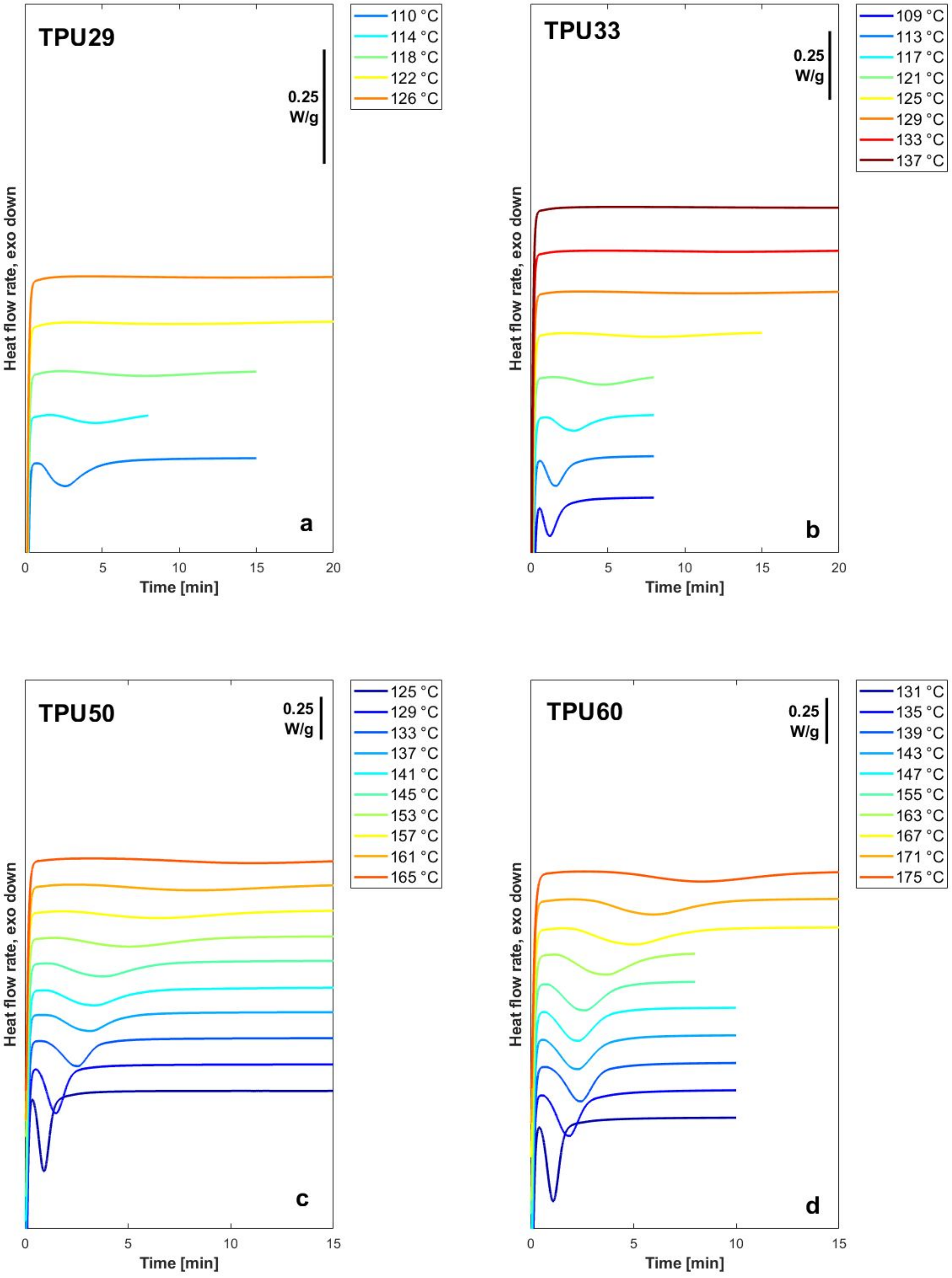

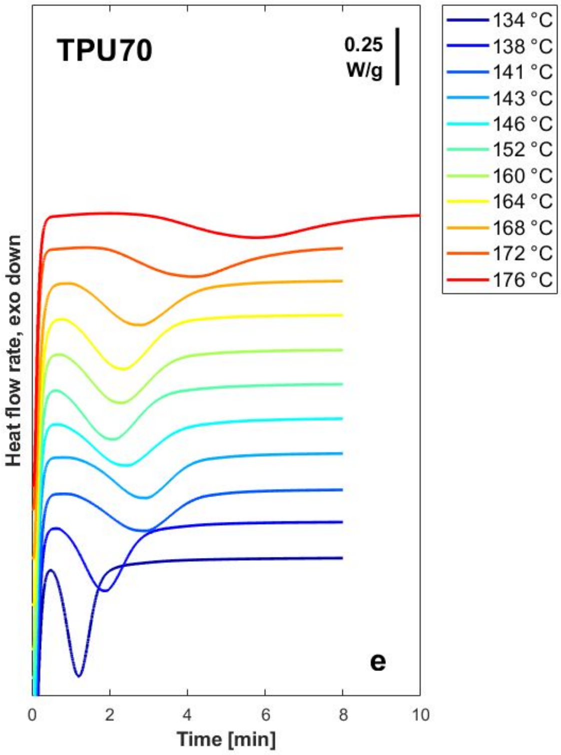

Figure S2. Representative DSC isothermal experiments showing the evolution of heat flow as a function of time at the different indicated crystallization temperatures for TPU29 (a), TPU33 (b), TPU50 (c), TPU60 (d), and TPU70 (e).

HS-content dependent crystallization kinetics

At first, the rate of crystallization in the low- and high-temperature "branches" of the curves of Figure 2 of the main text can be analyzed. The reciprocal of the crystallization peak time, evaluated at a temperature of 134 °C and 160 °C (see vertical lines in Figure 3), is reported as a function of the HS content in Figure 4a. By analogy with similar measurements in the literature,[1] as well as with the knowledge gathered in previous works on non-isothermal crystallization of these systems,[2,3] we tentatively attribute crystallization

at high and low supercooling to the development of the Form I and Form II polymorphs, respectively.

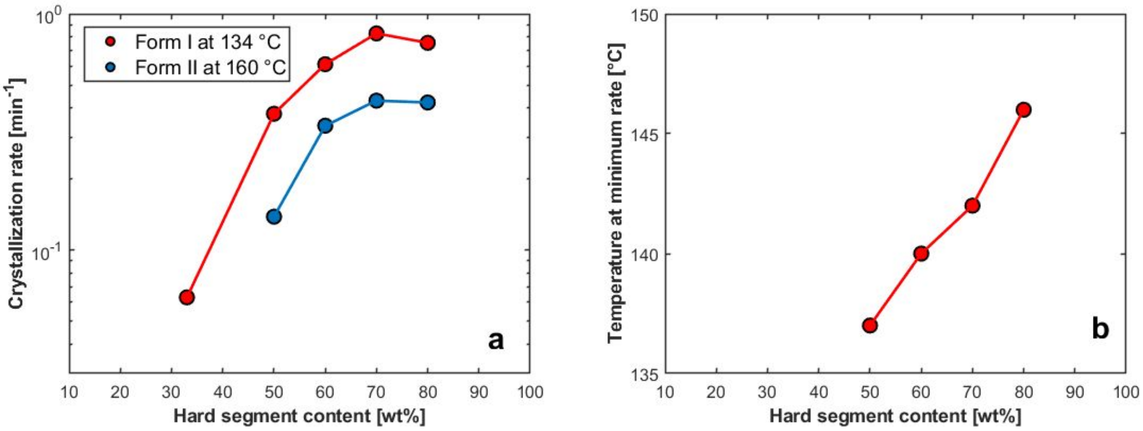

Figure S3. Rate of formation of the different polymorphs evaluated at 160 °C (Form II) and 134 °C (Form I) (a) and temperature at the minimum crystallization rate (b) versus hard segment content.

Therefore, under this hypothesis, Figure 4a shows that for both polymorphs, the kinetics of crystallization evaluated at a constant temperature accelerate with the increase of the weight percentage of hard segments. This increase, initially steep for low HS contents, tends to saturate at approximately 70 wt%. It is suggested that the observed effect might be due to a progressive decrease in the dilution of the crystallizing segments with increasing their concentration in the polymer.

Furthermore, for the samples that display the relative minimum in the overall crystallization rate, the temperature for its occurrence is reported as a function of HS content in Figure 4b. It is clear that this temperature increases with increasing the content of the crystallizing hard segments, from 137 °C at an HS of 50 wt% to about 10 K higher when the content of HS is 80 wt%. This change in the temperature where the minimum is located is possibly related to the shift in the melting temperature towards higher values with increasing HS content (not shown) and, more in general, to the different temperature regions for the

development of the two polymorphs, as evidenced in previous publications in the literature[1,4] and from our group.[2,3]

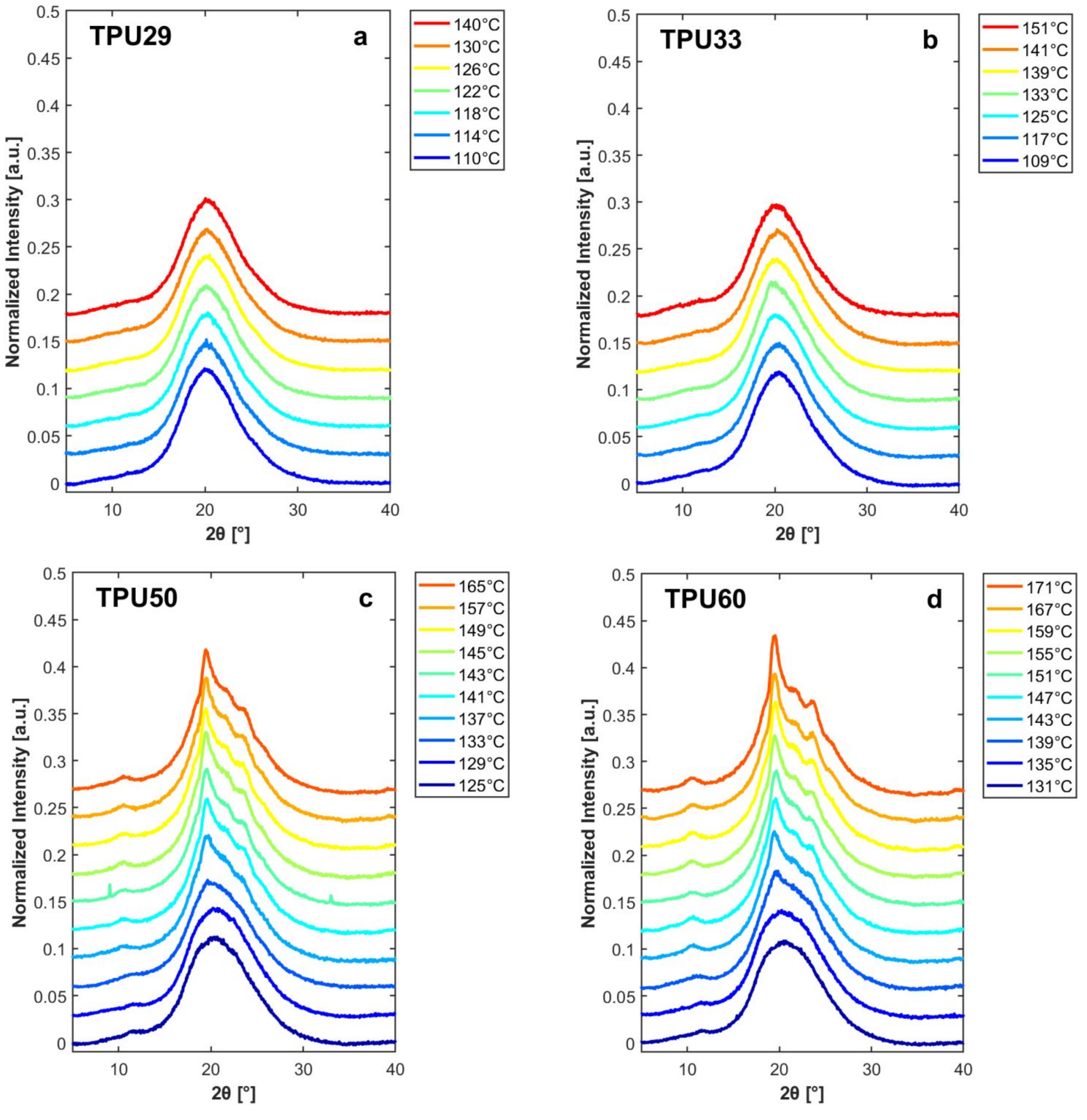

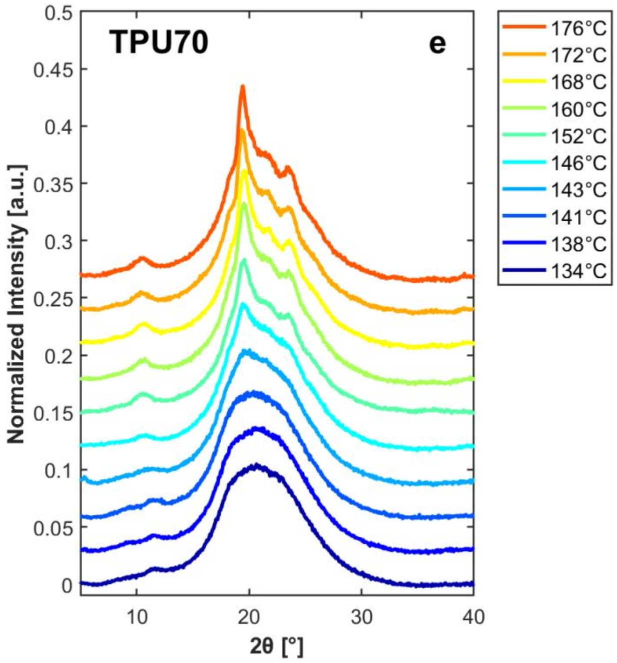

Figure S4. WAXD patterns recorded at room temperature after crystallizing TPU29 (a), TPU33 (b), TPU50 (c), TPU60 (d), and TPU70 (e) at the indicated crystallization temperatures.

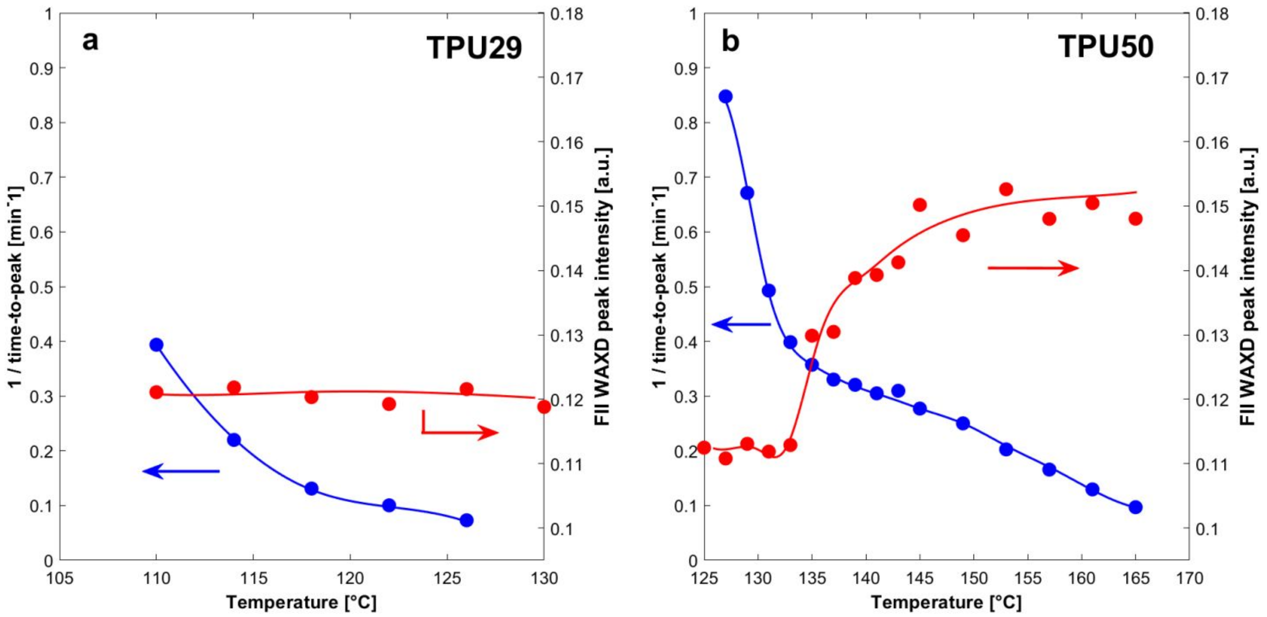

Figure S5. Comparison of reciprocal of crystallization peak time from DSC and Form II WAXD intensities at approximately 20° of 2θ, as a function of crystallization temperature for TPU29 (a), TPU50 (b). The continuos lines are a guide to the eyes.

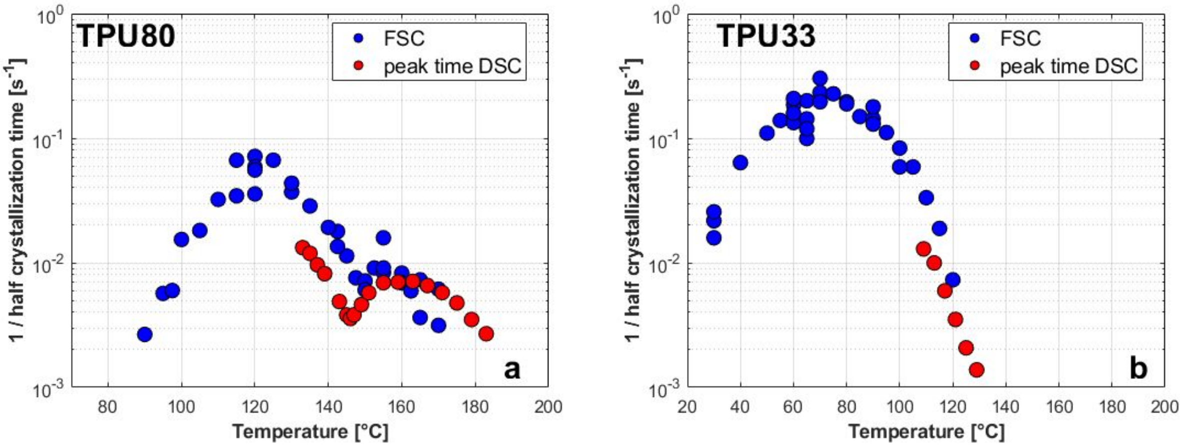

Figure S6. Comparison between the reciprocal of the half crystallization time measured by FSC and of the peak time measured by DSC as a function of crystallization temperature for TPU80 (a) and TPU33 (b).

REFERENCES

(1) Liu, F.; Liao, X.; Peng, Q.; Zhao, Y.; Li, S.; Li, G. Effect of Two Crystalline Forms on the Multiple Melting and Crystallization Kinetics of Thermoplastic Polyurethane. *Crystal Growth & Design* **2022**, *22* (10), 6015–6022. https://doi.org/10.1021/acs.cgd.2c00654.

(2) Baouch, Z.; Jariyavidyanont, K.; Moni, L.; Sangroniz, L.; Pöselt, E.; Müller, A.; Androsch, R.; Cavallo, D. Cooling Rate-Dependent Polymorphism in Thermoplastic Polyurethanes: Effect of Hard Segments Content. *Polymer* **2025**, *328*, 128477. https://doi.org/10.1016/j.polymer.2025.128477.

(3) Baouch, Z.; Sangroniz, L.; Shi, Y.; Pöselt, E.; Müller, A. J.; Cavallo, D. Self-Nucleation Enables Polymorphic Selection in Thermoplastic Polyurethanes. *Macromolecules* **2025**. https://doi.org/10.1021/acs.macromol.5c01477.

(4) Wang, Z.; Li, X.; Pöselt, E.; Eling, B.; Liao, T.; Wang, Z. Polymorphic Microstructure of MDI/BD-Block Polyurethane as Determined by Temperature-Sensitive Conformation Variation. *Soft Matter* **2021**, *17* (41), 9447–9456. https://doi.org/10.1039/D1SM01283E.
